# Supplementary material for: A set of multi-entry identification keys to African frugivorous flies (Diptera, Tephritidae)
Source: Zookeys. 2014 Jul 24;(428):97–108. doi: 10.3897/zookeys.428.7366 (PMC4143993; doi:10.3897/zookeys.428.7366)
Supplement: Supplementary material 6 — Key to Ceratitis [file zookeys-428-097-s006.zip › SF6_ZooKeys_key to Ceratitis/key/SF6_ZooKeys_key to Ceratitis/Media/Html/Ceratitis pinnatifemur .htm]

Ceratitis (Pterandrus) pinnatifemur Enderlein


***Ceratitis*** **(*Pterandrus*) *pinnatifemur*
Enderlein**

*Ceratitis* *pinnatifemur* Enderlein, 1920:
353.

Body
length: 5.05 (4.60-5.30) mm; wing length: 5.01 (4.40-5.55) mm.

Male

Head: Antenna yellowish orange. First flagellomere 2-3 times
as long as pedicel. Arista with short to moderately long rays; ventral rays
shorter and sparser than dorsal rays, especially basally. Frons yellow to
yellowish orange; with short scattered setulae distinctly darker than frons.
Frontal setae well developed. Face yellowish white; gena slightly darker. Genal
seta and setulae dark, well developed. Occiput dark brown on dorsal 0.33,
except along dorsal margin.

Thorax:
Postpronotal lobe white, with black or brownish spot. Scutal pattern: ground
color gray with golden shine; with streaks and darker markings but without
distinct spots, except shiny black sutural and dorsocentral spots; prescutellar
white markings separate. Scapular setae dark. Scutellum yellowish white,
basally with two separate dark spots, apically with three separate dark spots,
extending to basal 0.33. Anepisternum on ventral half brown; setulae pale
except few dark setulae across posterior margin on ventral half.

Legs:
Yellow except where otherwise noted; setation typical for subgenus, mainly
dark. Foreleg: femur posteriorly with dispersed bush of long dark setulae along
entire length, posterodorsal setulae longer; main area with pale brownish bushy
pilosity; ventral setae dark. Midleg: femur posteriorly completely dark,
anteroventrally dark brown on distal half, anterodorsally with tuft of black
appressed setae; ventrally with black feathering along distal 0.33. Hindleg:
femur at apical 0.25 with longer setulae dorsally and ventrally.

Wing:
bands brown. Marginal band forming continuous band with anterior part of discal
band; cubital band free; medial band absent; crossvein R-M opposite middle of
discal cell. Apex of vein R_1_ distal to level of crossvein R-M.
Crossvein DM-Cu oblique anterobasally.

Abdomen:
Mostly yellow. Tergites 1 and 3 across posterior part with continuous brown
band; tergites 2 and 4 on posterior half pale gray (not silvery); tergite 5
anteriorly broadly and posteriorly narrowly yellowish brown. Male epandrium in
lateral view with posterior lobe of surstylus short and slightly curved.

Female

As
male except for the following characters: antenna darker, first flagellomere
partly dark brown. Anepisternal pilosity with dark setulae extending somewhat
further anteriorly of hind margin. Legs without feathering, yellow with femora
orange-brown; forefemur posteroventrally with dark pilosity. Crossvein DM-Cu
oblique posterobasally. Ovipositor shorter than preabdomen. Aculeus at least 10
times as long as wide; tip distinctly broadened, and lateral margin straight.

(Description
after De Meyer & Freidberg, 2006)
